# Alteration of circulating microbiome and its associated regulation role in rheumatoid arthritis: Evidence from integration of multiomics data

**DOI:** 10.1002/ctm2.229

**Published:** 2020-11-16

**Authors:** Xing‐Bo Mo, Chen‐Yue Dong, Pei He, Long‐Fei Wu, Xin Lu, Yong‐Hong Zhang, Hong‐Wen Deng, Fei‐Yan Deng, Shu‐Feng Lei

**Affiliations:** ^1^ Center for Genetic Epidemiology and Genomics School of Public Health Medical College of Soochow University Suzhou Jiangsu P. R. China; ^2^ Jiangsu Key Laboratory of Preventive and Translational Medicine for Geriatric Diseases Soochow University Suzhou Jiangsu P. R. China; ^3^ Department of Epidemiology School of Public Health Medical College of Soochow University Suzhou Jiangsu P. R. China; ^4^ Center of Bioinformatics and Genomics Department of Global Biostatistics and Data Science Tulane University New Orleans Louisiana

To the Editor:

Rheumatoid arthritis (RA) is a chronic systemic autoimmune disease characterized by symmetric polyarthritis and the presence of autoantibodies. Recent studies have shown that blood is not as sterile as previously supposed,^1^, ^2^ and blood microbial dysbiosis is implicated in the pathogenesis of some diseases,^2^, ^3^ but not reported in RA.

Our previously identified RA‐associated interferon‐inducible gene network has strongly implicated that the circulation microbial dysbiosis probably is served as an important RA‐associated environmental factor.^4^ To identify the alterations of circulating microbiome associated with RA and its regulation role, we generated and integrated three omics datasets (microbiome, methylome, and transcriptome) from the same subjects, described the composition and richness of blood bacteria between RA cases and controls, identified significant RA‐associated taxa, and performed in‐depth correlation analysis and causal inference test to evaluate regulation role of the detected bacteria community on the pathogenesis of RA.

A total of 28 female patients and 15 age‐ and sex‐matched controls (Table S1) were enrolled according to several steps detailed in the Supporting Information. We determined blood microbiome by bacterial 16S ribosomal DNA sequencing. The 1352 OTUs detected could be classified into 12 phyla, 26 classes, 54 orders, 115 families, and 216 genera (Table S2). Among the 10 known phyla, *Proteobacteria*, *Actinobacteria*, *Bacteroidetes*, *Candidatus Saccharibacteria*, and *Firmicutes* have average relative abundances of 77.56% (predominated), 13.23%, 3.98%, 3.05%, and 1.28%, respectively (Table S3). This result was consistent with previous findings from healthy individuals.^1^

No significant *α*‐diversity was detected between the two groups (Figure 1A),^5^ but significant major separation in *β*‐diversity was observed between RA patients and the controls (Figure 1B). Compared to the controls, the microbiome of RA blood showed significantly higher mean distances in PCoA1 axis, but no significant difference in PCoA2 axis (Figure 1C).

**FIGURE 1:**
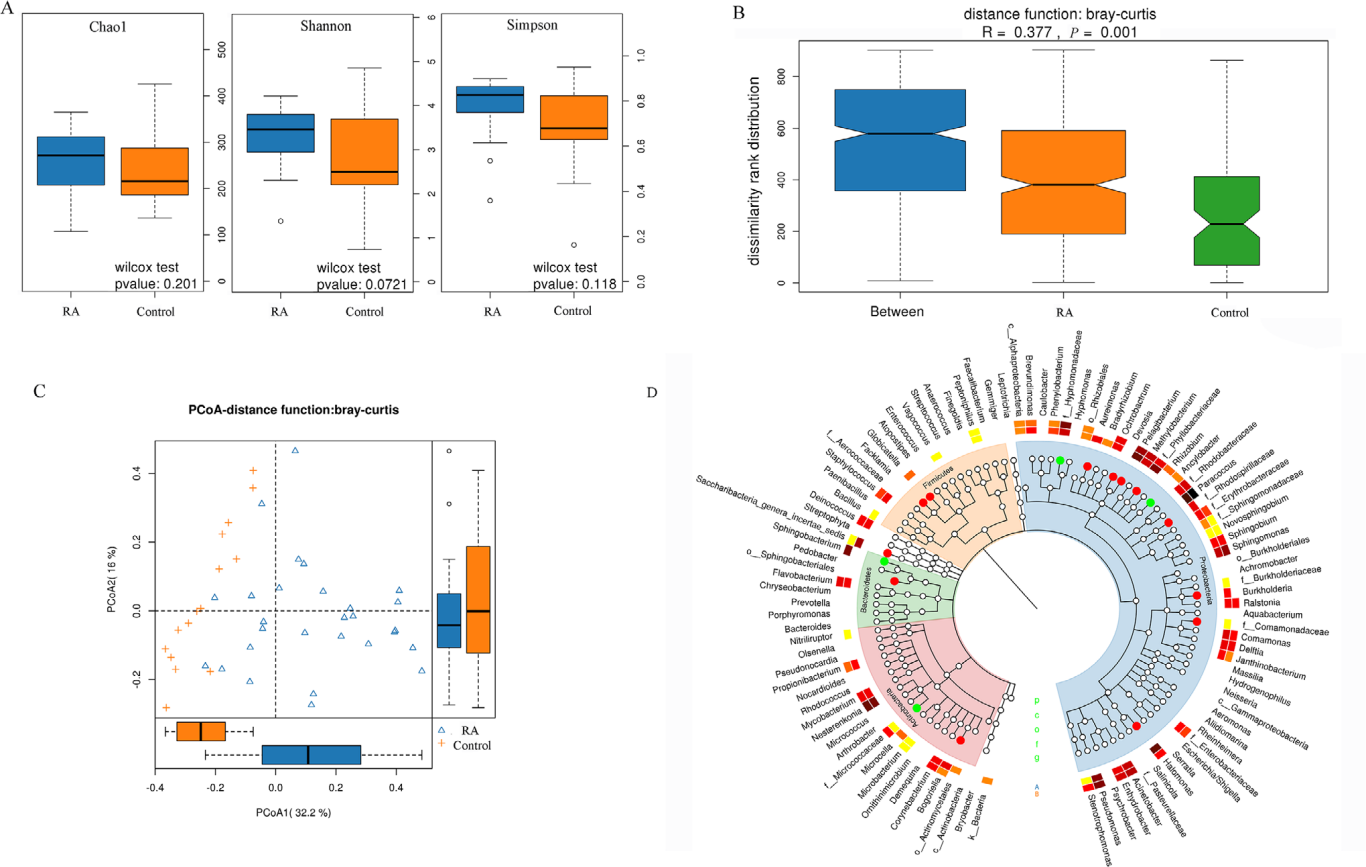
Comparison of the *α*‐diversity and *β*‐diversity of the blood microbiome in the rheumatoid arthritis (RA) cases and controls. A, We measured the *α*‐diversity of bacterial community in each group using three indices (eg, Chao 1, Shannon, and Simpson), which considered both community richness and evenness. B, ANOSIM test (*P* = .001) for the within‐group and between‐group variance. C, Principal coordinates analysis (PCoA) directly and completely present bacterial flora distribution between communities. PCoA plot generated based on Bray‐Curtis distances. The *x*‐ and *y*‐axes indicate the first and second coordinates, respectively, and the values in parentheses show the percentages of the community variation explained. D, The inner circle represents the classification level from phylum to genus. Each small circle at a different classification level represents a classification at that level, and the diameter of the small circle represents the relative abundance. The significantly different species are colored according to the groups. The red nodes represent the microbial groups enriched in the RA group and the green nodes represent the microbial groups enriched in the control group. The middle layer is the heatmap of the mean abundance. The darker the color, the higher it is; the outermost layer is the species annotation

Linear discriminant analysis effect size (LEfSe) algorithm^6^ was used to compare bacterial abundances at different taxonomic levels (five phyla, 14 classes, 37 orders, 97 families, and 196 genera) (Figure 1D). Some bacterial taxa (two phyla, three classes, six orders, 10 families, and 13 genera) have significant differences in abundance between the RA cases and controls (Table S4), for example, *Bacteroidetes* (0.0126 ± 0.0200 vs 0.0669 ± 0.0341, FDR *q*‐value = 3.51 × 10^−7^) and *Candidatus Saccharibacteria* (0.0605 ± 0.1137 vs 0.0006 ± 0.0023, FDR *q*‐value = 1.08 × 10^−4^) (Table S3). When compared our results with the findings from other studies focused on gut and synovial fluid microbiome, both consistent and inconsistent results exist. For example, the lower abundance of *Bacteroidetes* in the blood was consistent with that in the synovial fluid,^7^ but inconsistent with that in the gut of RA patients.^8^ *Proteobacteria* are one of the most abundant phyla in human microbiota. The proportion of *Proteobacteria* in the synovial fluid of RA patients was higher than that of controls.^7^ Our results consistently suggested that the prevalence of genera *Pelagibacterium*, *Halomonas*, *Aureimonas*, *Chelativorans*, and others that belong to the *Proteobacteria* phylum was higher in RA than the controls.

These results taken together showed that alterations of circulating microbiome were associated with RA. In addition, the differences in microbiomes of RA patients and controls suggested some bacteria‐derived functions, including cellular processes, environmental information processing, genetic information processing (transcription and translation), and metabolism (Table S5).

Peripheral blood mononuclear cells (PBMCs) containing monocytes and lymphoid cells represent circulating mixture cells directly involved in autoimmunity and chronic inflammation. It is intuitively inferred that PBMCs and circulating microbiota live in the same environment of peripheral blood, and they can directly contact and have potential effective interactions.^9^, ^10^ Therefore, we generated the mRNA expressions and DNA methylation data in PBMCs from the same sample set by using the Human Gene Expression Microarray V.4.0 (CaptialBio, Beijing, China) and Illumina 450 K Infinium Methylation BeadChip (Illumina, Inc., USA), respectively.

To detect the potential regulation roles of the identified microbial dysbiosis, we integratively analyzed the three omics data (microbiome, methylome, and transcriptome). We first performed differential expression analyses, and a total of 47 DNA methylations (|Δ*β*| > .05 and *P* < 5.0 × 10^−4^) and 749 mRNAs (fold change > 2, *P* < 5.0 × 10^−4^) were selected. As expected, we detected significant correlations between seven taxa within *Proteobacteria* phylum and DNA methylation (Table S6), and the DNA methylation site cg00959259 in *PARP9* was highlighted as its consistent associations with three taxa of order *Rhizobiales*, family *Hyphomicrobiaceae*, and genus *Pelagibacterium*, with spearman correlation coefficients of −.6070 (*P* = 1.66 × 10^−3^), −.5539 (*P* = 4.98 × 10^−3^), and −.5626 (*P* = 4.21 × 10^−3^), respectively (Figure 2).

**FIGURE 2:**
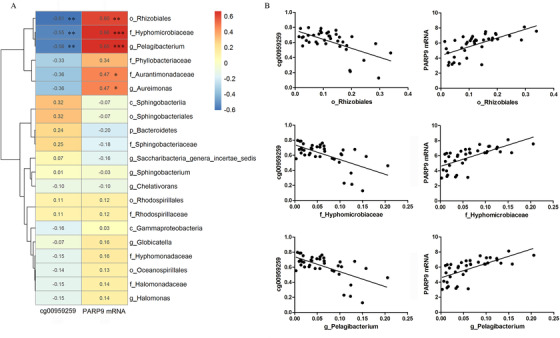
Associations between blood bacteria and methylation and mRNA levels of *PARP9* in peripheral blood mononuclear cells (PBMCs). A, Heatmap of Spearman correlations between blood bacteria and methylation and mRNA levels of *PARP9* in PBMCs. Numbers in the grids are Spearman correlation coefficients (*P < .05, **P < .01, ***P < .001). B, Scatter plots show the relationships of order *Rhizobiales*, family *Hyphomicrobiaceae*, and genus *Pelagibacterium* with DNA methylation of CpG site cg00959259 and *PARP9* mRNA levels in PBMCs. These three taxa belong to the *Proteobacteria* phylum, family *Hyphomicrobiaceae* belongs to order *Rhizobiales*, and genus *Pelagibacterium* belongs to family *Hyphomicrobiaceae*

For mRNA expression, the microbial abundances in order *Rhizobiales*, family *Hyphomicrobiaceae*, and genus *Pelagibacterium* were significantly associated with *PARP9* mRNA level, with spearman correlation coefficients of .6040 (*P* = 1.77 × 10^−3^), .6632 (*P* = 4.12 × 10^−4^), and .6549 (*P* = 5.15 × 10^−4^), respectively (Figure 2; Table S7). Through causal inference test, we detected causal effects of order *Rhizobiales*, family *Hyphomicrobiaceae*, and genus *Pelagibacterium*, and the mediation effect of *PARP9* mRNA expression on RA risk (Table S8). Previously, we had shown that lower cg00959259 methylation level and higher *PARP9* mRNA expression level in PBMCs were associated with higher RA risk.^4^ Therefore, by combining the current results with our previously reported results,^4^ it seems that blood bacteria within the *Proteobacteria* phylum may increase RA risk through demethylation of *PARP9*, which may lead to higher expression of the *PARP9* gene in PBMCs. These observations suggested that the identified microbiome alterations may have regulatory effects on gene expressions.

In summary, this was the first study identifying significant alterations in blood microbiome in RA, detecting significant associations between blood bacteria and gene expressions in PBMCs, and highlighting the regulatory effects of circulating bacterial community on RA risk through the mediation of the expression of *PARP9*. These findings enhanced our understanding of the roles of circulating microbiome in the pathology of RA. These results suggested that the detection of imbalances in microbial composition could facilitate early diagnosis of RA, and bacterial stimuli or control could be developed as useful microbiome‐based strategies for RA prevention and treatment.

## CONFLICT OF INTEREST

The authors declare that there is no conflict of interest.

## ETHICS STATEMENT

The study protocol was approved by ethical committees of Soochow University. All study participants provided their written consent for participation in the study.

## AUTHOR CONTRIBUTIONS

Xing‐Bo Mo, Chen‐Yue Dong, Pei He, Long‐Fei Wu, and Xin Lu recruited the patients and conducted the experiments. Xing‐Bo Mo, Chen‐Yue Dong, and Yong‐Hong Zhang drafted and revised the manuscript. Xing‐Bo Mo and Chen‐Yue Dong analyzed the data. Hong‐Wen Deng, Fei‐Yan Deng, and Shu‐Feng Lei designed and supervised the study, and revised the manuscript. All the authors read and approved the final manuscript.

## Supporting information

Supporting Information

## Data Availability

The datasets used and/or analyzed during the current study are available from the corresponding author upon reasonable request.
