# Supplementary material for: Alteration of circulating microbiome and its associated regulation role in rheumatoid arthritis: Evidence from integration of multiomics data
Source: Clin Transl Med. 2020 Nov 16;10(7):e229. doi: 10.1002/ctm2.229 (PMC7668190; doi:10.1002/ctm2.229)
Supplement: Supplementary file 1 — Supporting Information [file CTM2-10-e229-s001.docx]

**Supplementary information**

**Materials and Methods**

**Subjects**

RA patients attending the Rheumatology Clinic at the First Affiliated Hospital of Soochow University that fulfilled the exclusion and inclusion criteria were asked to enroll in the study. A total of 28 female patients who met the 2010 American College of Rheumatology/European League Against Rheumatism (ACR/EULAR) criteria[^1^](#_ENREF_1) for RA were recruited (**Supplementary Table S1**). Disease activity, which was defined as an EULAR Disease Activity Score (DAS28),[^2^](#_ENREF_2) of the 28 patients ranged from 2.91 to 6.41. A total of 15 female subjects without RA were recruited as controls. We have adopted some steps to select the subjects. First, all the subjects, including controls and RA cases, were from a similar source population (Han Chinese individuals living in Suzhou city of the Jiangsu province); Second, age and sex were two RA-associated factors used in matching the subjects. All the studied subjects were female and the age has no statistical difference between the cases and controls (**Table S1**); Third, to exclude the confounding effects of other diseases we also exclude any RA patient or controls with the following diseases and/or conditions: taking antibiotics, consuming probiotics, or having a known history of inflammatory bowel disease or other autoimmune diseases like systemic lupus erythematosus, diabetes and multiple sclerosis. The study was approved by the ethical committee of Soochow University (No: 2012-146). The written informed consent was obtained from all the subjects.

**Biological sample collection**

Peripheral blood (15 ml for each participant) was collected by venipuncture and stored in sodium citrate supplemented vacuum tubes. PBMCs were isolated by density gradient centrifugation at room temperature at 3000 g for 10 minutes using Lymphoprep (Sigma, life science, USA) within 4 hours after phlebotomy, and then treated with TRIzol reagent (invitrogen, Carlsbad, California, USA) and stored at -80℃ to avoid RNA degradation. The plasma samples were frozen at -80°C for quick preservation. Total RNA and DNA were extracted from PBMCs according to the instructions recommended by the manufacture and then quantified by using NanoDrop ND-1000 (Thermo Scientific, Wilmington, Delaware, USA) spectrophotometer.

**DNA extraction, PCR amplification, and sequencing**

DNA separation and PCR were performed in laminar air flow platform, and UV lamps were used before starting to avoid possible contaminants. Blood bacterial genomic DNA was isolated from plasma sample. 16S ribosomal DNA gene high-throughput sequencing using a molecular pipeline specifically optimized for blood samples was utilized to compare the bacterial community composition of peripheral blood in RA cases and controls. The v3-v4 region of 16S ribosomal DNA gene was amplified by PCR using forward primers (5'-cctacgggnggcwgcag) and reverse primer (5'-GACTACHVGGGTATCTAATCC) by Qiagen's QIAmp DNA kit (Qiagen Inc., Germantown, MD, USA). Each reaction contains a blank a negative control with no bacteria for quality confirmation. Moreover, every board was sequenced in a MiSeq operation, including two repeated quality control and a negative control samples (only pyrolysis buffer and kit reagent) which would expand standardization, consolidation. The genes were extracted, amplified, constructed to library and tested on the Illumina MiSeq instrument using a MiSeq Reagent Kit PE300 v3 kit.

**Derivation of microbiome data**

QIIME package (quantitative analysis of microbial ecology, QIIME: http://qiime.org/) v1.8 was used for sequence reading and select low-quality readings with scores less than 30. In addition, the chimeric sequence was removed. The diversity and richness of bacteria in the blood samples of the processed sequences were calculated using several estimates. OTUs were clustered at a 97% similarity cut-off value. The Silva database (Release128, http://www.arb-silva.de) was used to obtain specific taxonomic information corresponding to each OTU. α and β diversities were compared between the two groups. α diversity measures the species richness within each sample from the rarefied OTU table. We used the Chao1, Shannon and Simpson indices that determine the diversity from the abundance data (observed taxa) and an estimation of the unobserved taxa (unobserved rare taxa).[^3^](#_ENREF_3) β diversity allows for comparison of the taxonomic profiles between pairs of individual samples. We measured the β diversity looking at the OTU distribution of each sample using the Bray-Curtis distances. Bacterial gene functions were predicted from 16S ribosomal DNA gene-based microbial compositions using the PICRUSt algorithm to make inferences from the KEGG annotated databases. In this study, we limited the analysis of phyla of the bacterial to populations with mean relative abundance ≥ 1%. For lower level taxa (classified as genera), we limit our analysis to taxa with mean relative abundance ≥ 0.01%.

**Genome-wide expression profiling**

DNA methylation and mRNA expression profiling in the 28 female patients and 15 controls have been described previously.[^4^](#_ENREF_4) Briefly, DNA methylation levels were measured using Illumina 450K Infinium Methylation BeadChip (Illumina, Inc., USA) according to the manufacturer’s instructions. The methylation level of a locus was measured as β = M/(M + U) where M was the methylated signals and U was the methylation level of the unmethylated signals. This methylation value β continuously ranges from 0 (unmethylated) to 1 (fully methylated).

Genome-wide mRNA expression was profiled using Human Gene Expression Microarray V4.0 (CapitalBio Corp, Beijing, China) according to the manufacturer’s instructions. The data was extracted by Agilent Feature Extraction (V10.7) and was summarized, normalized and controlled for quality using GeneSpring GX program (V12.0). Then, log_2_ transformation was applied to the data using the Adjust Data function of Multi experiment Viewer software. Probes with detection rate less than 80% and/or incomplete annotation information were filtered out.

**Statistical analyses**

When comparing the variables among groups, Student's *t* test, Wilcoxon rank sum test, chi-squared test or Fisher exact test was used as appropriate. Linear discriminant analysis effect size (LEfSe) algorithm was used to measure OTU differences between groups.[^5^](#_ENREF_5) The α-diversity was determined by the Wilcoxon rank sum test. The analysis of similarities (ANOSIM) test was applied to test the β-diversity based on the Bray-Curtis distance matrix containing all analyzed samples in order to define if the overall structure of the microbiota was significantly different between the different groups. Principal coordinates analysis (PCoA) directly and completely present bacterial flora distribution between communities. Multiple test correction was performed using the Benjamini and Hochberg false discovery rate (FDR).[^6^](#_ENREF_6) Inferred microbiome functions based on 16S rRNA gene sequences were also explored using the PICRUSt (phylogenetic investigation of communities by reconstruction of unobserved states) algorithm, a computational approach to predict the functional composition of a metagenome using marker gene data and a database of reference genomes.[^7^](#_ENREF_7) Correlations between 16S ribosomal DNA quantitative levels, DNA methylation and mRNA levels in PBMCs were calculated using Spearman rank order correlation. The relationship of 16S ribosomal DNA quantitative levels, DNA methylation or mRNA levels and RA risk were further assessed by causal inference test to test the causal effect of bacteria and the mediation effect of methylation or mRNA expression on RA risk.[^8^](#_ENREF_8)^,^ [^9^](#_ENREF_9) SAS 9.4 (SAS Institute, Cary, NC, USA) and R (https://www.r-project.org/) softwares were used for statistical analysis.

**References**

1. Aletaha D, Neogi T, Silman AJ, et al. 2010 Rheumatoid arthritis classification criteria: an American College of Rheumatology/European League Against Rheumatism collaborative initiative. *Arthritis Rheum.* 2010; 62: 2569-81.

2. Prevoo ML, van 't Hof MA, Kuper HH, van Leeuwen MA, van de Putte LB, van Riel PL. Modified disease activity scores that include twenty-eight-joint counts. Development and validation in a prospective longitudinal study of patients with rheumatoid arthritis. *Arthritis Rheum.* 1995; 38: 44-8.

3. Hughes JB, Hellmann JJ, Ricketts TH, Bohannan BJ. Counting the uncountable: statistical approaches to estimating microbial diversity. *Appl Environ Microbiol.* 2001; 67: 4399-406.

4. Zhu H, Wu LF, Mo XB, et al. Rheumatoid arthritis-associated DNA methylation sites in peripheral blood mononuclear cells. *Ann Rheum Dis.* 2019; 78: 36-42.

5. Segata N, Izard J, Waldron L, et al. Metagenomic biomarker discovery and explanation. *Genome Biol.* 2011; 12: R60.

6. Glickman ME, Rao SR, Schultz MR. False discovery rate control is a recommended alternative to Bonferroni-type adjustments in health studies. *J Clin Epidemiol.* 2014; 67: 850-7.

7. Langille MG, Zaneveld J, Caporaso JG, et al. Predictive functional profiling of microbial communities using 16S rRNA marker gene sequences. *Nat Biotechnol.* 2013; 31: 814-21.

8. Millstein J, Zhang B, Zhu J, Schadt EE. Disentangling molecular relationships with a causal inference test. *BMC Genet.* 2009; 10: 23.

9. Liu Y, Aryee MJ, Padyukov L, et al. Epigenome-wide association data implicate DNA methylation as an intermediary of genetic risk in rheumatoid arthritis. *Nat Biotechnol.* 2013; 31: 142-7.

**Table S1 Baseline characteristics of the study subjects**

|  | **RA(n = 28)** | **Control(n = 15)** | ***P* value** |
| --- | --- | --- | --- |
| Gender (female) | 28 | 15 | - |
| Age (years) | 44.99±9.45 | 50.48±14.05 | 0.14 |
| Body mass index (kg/m^2^) | 20.35±2.99 | 22.57±2.33 | 0.84 |
| RF positive rate(%) | 11/15(73%) | NA | - |
| WBC(10^9^/L) | 7.54±2.45 | NA | - |
| LY(10^9^/L) | 3.27±5.99 | NA | - |
| CRP(mg/L) | 13.93±16.61 | NA | - |
| NSAID(%) | 22/28(79%) | NA | - |
| Glucocorticoid(%) | 19/28(68%) | NA | - |
| Antirheumatic(%) | 24/28(86%) | NA | - |

Results expressed as mean ± standard error or number of patients (%).

RA patients attending the Rheumatology Clinic at the First Affiliated Hospital of Soochow University that fulfilled the exclusion and inclusion criteria were asked to enroll in the study. A total of 28 female patients who met the 2010 American College of Rheumatology/European League Against Rheumatism (ACR/EULAR) criteria for RA were recruited. Disease activity, which was defined as an EULAR Disease Activity Score (DAS28), of the 28 patients ranged from 2.91 to 6.41. A total of 15 female subjects without RA were recruited as controls. Any patient or control on antibiotics, consuming probiotics, or having a known history of inflammatory bowel disease or other autoimmune diseases like systemic lupus erythematosus, diabetes and multiple sclerosis were excluded.

**Table S2 Sequencing data of blood microbes in RA case and control groups**

| Groups | Raw sequences | Superior sequences | OTU number | Numbers of different classification orders | | | | |
| --- | --- | --- | --- | --- | --- | --- | --- | --- |
|  |  |  |  | Phylum | Class | Order | Family | Genus |
| RA | 1003867 | 975041 | 1150 | 12 | 25 | 50 | 104 | 195 |
| Control | 558839 | 542477 | 709 | 11 | 20 | 41 | 83 | 133 |
| Total | 1562706 | 1517518 | 1352 | 12 | 26 | 54 | 115 | 216 |

The total number of reads obtained from 43 participants was 1,562,706. After filtering and removing the chimeric sequences, we obtained 975,041 high-quality sequences from the RA patients and 542,477 sequences from the controls.

**Table S3 Relative abundances of blood bacterial taxa between RA cases and controls at the phylum level**

| **Phylum** | **Average** | **RA** | **Control** | ***P* value** | **q value** |
| --- | --- | --- | --- | --- | --- |
| Proteobacteria | 0.7756 | 0.8102±0.1378 | 0.7409±0.1657 | 7.58E-02 | 1.84E-01 |
| Actinobacteria | 0.1323 | 0.0891±0.0693 | 0.1754±0.1642 | 4.52E-02 | 2.71E-01 |
| Bacteroidetes | 0.0398 | 0.0126±0.0200 | 0.0669±0.0341 | 2.93E-08 | 3.51E-07 |
| Candidatus Saccharibacteria | 0.0305 | 0.0605±0.1137 | 0.0006±0.0023 | 9.02E-06 | 1.08E-04 |
| Firmicutes | 0.0128 | 0.0188±0.0327 | 0.0068±0.0094 | 2.52E-02 | 1.01E-01 |
| Deinococcus-Thermus | 0.0067 | 0.0059±0.0081 | 0.0074±0.0101 | 2.77E-01 | 4.74E-01 |
| Cyanobacteria/Chloroplast | 0.0007 | 0.0006±0.0011 | 0.0008±0.0024 | 7.65E-02 | 1.84E-01 |
| Fusobacteria | 0.0004 | 0.0001±0.0003 | 0.0007±0.0025 | 2.64E-01 | 9.03E-01 |
| Acidobacteria | 0.0003 | 0.0006±0.0026 | 0.0000±0.0000 | 4.27E-01 | 7.87E-01 |
| Tenericutes | 0.0000 | 0.0000±0.0001 | 0.0000±0.0000 | 1.02E-01 | 2.04E-01 |

The present data revealed the decreased abundance of *Bacteroidetes* phylum (0.0126±0.0200 vs. 0.0669±0.0341, FDR q value = 3.51× 10^-7^) in RA patients than controls. The *Candidatus Saccharibacteria* (0.0605±0.1137 vs. 0.0006±0.0023, FDR q value = 1.08× 10^-4^) phylum was significantly higher in RA group than controls.

**Table S4 Relative abundances of significant blood bacterial taxa between RA cases and controls**

| **Bacteria ^#^** | **RA** | **Control** | ***P* value** | **Q value** |
| --- | --- | --- | --- | --- |
| p_Actinobacteria; c_Actinobacteria; o_Actinomycetales; f_Bogoriellaceae | 0.0044±0.0045 | 0.0010±0.0015 | 1.69E-03 | 2.43E-02 |
| p_Actinobacteria; c_Actinobacteria; o_Actinomycetales; f_Bogoriellaceae; g_Bogoriella | 0.0044±0.0045 | 0.0010±0.0015 | 1.69E-03 | 3.04E-02 |
| p_Bacteroidetes | 0.0126±0.0200 | 0.0669±0.0341 | 2.93E-08 | 3.51E-07 |
| p_Bacteroidetes; c_Sphingobacteriia | 0.0066±0.0202 | 0.0543±0.0230 | 4.54E-07 | 1.18E-05 |
| p_Bacteroidetes; c_Sphingobacteriia; o_Sphingobacteriales | 0.0066±0.0202 | 0.0543±0.0230 | 4.54E-07 | 2.45E-05 |
| p_Bacteroidetes; c_Sphingobacteriia; o_Sphingobacteriales; f_Sphingobacteriaceae | 0.0060±0.0196 | 0.0543±0.0230 | 3.28E-07 | 3.77E-05 |
| p_Bacteroidetes;c_Sphingobacteriia;o_Sphingobacteriales;f_Sphingobacteriaceae;g_Sphingobacterium | 0.0058±0.0197 | 0.0533±0.0232 | 3.08E-07 | 6.50E-05 |
| p_Candidatus Saccharibacteria | 0.0605±0.1137 | 0.0006±0.0023 | 9.02E-06 | 1.08E-04 |
| p_Candidatus Saccharibacteria; c_undefined; g_Saccharibacteria genera incertae sedis | 0.0605±0.1137 | 0.0006±0.0023 | 9.02E-06 | 2.78E-04 |
| p_Firmicutes; c_Bacilli | 0.0156±0.0296 | 0.0036±0.0063 | 5.03E-03 | 4.36E-02 |
| p_Firmicutes; c_Bacilli; o_Bacillales | 0.0046±0.0048 | 0.0013±0.0030 | 1.75E-03 | 1.89E-02 |
| p_Firmicutes; c_Bacilli; o_Lactobacillales; f_Aerococcaceae | 0.0041±0.0088 | 0.0008±0.0030 | 7.47E-04 | 1.23E-02 |
| p_Firmicutes; c_Bacilli; o_Lactobacillales; f_Aerococcaceae; g_Facklamia | 0.0013±0.0030 | 0 | 3.22E-03 | 4.34E-02 |
| p_Firmicutes; c_Bacilli; o_Lactobacillales; f_Aerococcaceae; g_Globicatella | 0.0028±0.0062 | 0 | 3.44E-05 | 9.28E-04 |
| p_Proteobacteria; c_Alphaproteobacteria; o_Caulobacterales | 0.0442±0.0718 | 0.0586±0.0330 | 1.26E-03 | 3.41E-02 |
| p_Proteobacteria; c_Alphaproteobacteria; o_Caulobacterales; f_Hyphomonadaceae | 0.0117±0.0135 | 0.0557±0.0336 | 1.29E-06 | 7.40E-05 |
| p_Proteobacteria; c_Alphaproteobacteria; o_Rhizobiales | 0.1575±0.0715 | 0.0591±0.0385 | 8.42E-07 | 2.27E-05 |
| p_Proteobacteria; c_Alphaproteobacteria; o_Rhizobiales; f_Aurantimonadaceae | 0.0089±0.0161 | 0 | 4.12E-06 | 1.35E-04 |
| p_Proteobacteria; c_Alphaproteobacteria; o_Rhizobiales; f_Aurantimonadaceae; g_Aureimonas | 0.0089±0.0161 | 0 | 4.12E-06 | 1.69E-04 |
| p_Proteobacteria; c_Alphaproteobacteria; o_Rhizobiales; f_Hyphomicrobiaceae | 0.0876±0.0454 | 0.0213±0.0158 | 1.44E-07 | 1.65E-05 |
| p_Proteobacteria; c_Alphaproteobacteria; o_Rhizobiales; f_Hyphomicrobiaceae; g_Devosia | 0.0014±0.0032 | 0 | 1.97E-03 | 3.09E-02 |
| p_Proteobacteria; c_Alphaproteobacteria; o_Rhizobiales; f_Hyphomicrobiaceae; g_Pelagibacterium | 0.0862±0.0450 | 0.0212±0.0158 | 9.32E-08 | 2.01E-05 |
| p_Proteobacteria; c_Alphaproteobacteria; o_Rhizobiales; f_Phyllobacteriaceae | 0.0432±0.0256 | 0.0128±0.0184 | 8.98E-05 | 1.72E-03 |
| p_Proteobacteria; c_Alphaproteobacteria; o_Rhizobiales; f_Phyllobacteriaceae; g_Chelativorans | 0.0001±0.0001 | 0 | 3.12E-06 | 1.68E-04 |
| p_Proteobacteria; c_Alphaproteobacteria; o_Rhizobiales; f_Phyllobacteriaceae; g_Hoeflea | 0.0002±0.0003 | 0 | 3.98E-04 | 7.82E-03 |
| p_Proteobacteria; c_Alphaproteobacteria; o_Rhizobiales; f_Xanthobacteraceae | 0 | 0.0016±0.0035 | 1.42E-05 | 5.46E-04 |
| p_Proteobacteria; c_Alphaproteobacteria; o_Rhizobiales; f_Xanthobacteraceae; g_Ancylobacter | 0 | 0.0016±0.0035 | 1.42E-05 | 1.03E-03 |
| p_Proteobacteria; c_Alphaproteobacteria; o_Rhodospirillales | 0.0213±0.0435 | 0 | 8.38E-06 | 1.22E-04 |
| p_Proteobacteria; c_Alphaproteobacteria; o_Rhodospirillales; f_Rhodospirillaceae | 0.0213±0.0435 | 0 | 4.69E-06 | 1.35E-04 |
| p_Proteobacteria; c_Betaproteobacteria; o_Burkholderiales; f_Burkholderiaceae; g_Burkholderia | 0.0021±0.0031 | 0.0002±0.0005 | 2.00E-03 | 3.09E-02 |
| p_Proteobacteria; c_Gammaproteobacteria | 0.2258±0.1750 | 0.1073±0.0803 | 1.82E-03 | 2.37E-02 |
| p_Proteobacteria; c_Gammaproteobacteria; o_Oceanospirillales | 0.1782±0.1950 | 0.0035±0.0054 | 3.56E-07 | 1.92E-05 |
| p_Proteobacteria; c_Gammaproteobacteria; o_Oceanospirillales; f_Halomonadaceae | 0.1780±0.1951 | 0.0035±0.0054 | 6.80E-07 | 3.91E-05 |
| p_Proteobacteria; c_Gammaproteobacteria; o_Oceanospirillales; f_Halomonadaceae; g_Halomonas | 0.1779±0.1950 | 0.0030±0.0041 | 5.26E-07 | 5.68E-05 |

^#^ ‘p_’ represented phylum; ‘c_’ represented class; ‘o_’ represented order; ‘f_’ represented family; ‘g_’ represented genus.

On looking deeper within the *Bacteroidetes* phylum, the abundance of class *Sphingobacteriia*, order *Sphingobacteriales*, family *Sphingobacteriaceae*, and genus *Sphingobacterium* were significantly lower in RA patients than controls. In fact, order *Sphingobacteriales* belongs to class *Sphingobacteriia*, family *Sphingobacteriaceae* belongs to order *Sphingobacteriales*, and genus *Sphingobacterium* belongs to family *Sphingobacteriaceae*. Within the *Candidatus Saccharibacteria* phylum, only genus *Saccharibacteria genera incertae sedis* was detected to be significantly higher in RA group. *Firmicutes* was not significantly different between RA cases and controls at phylum level, but class *Bacilli*, order *Bacillales*, family *Aerococcaceae*, genus *Facklamia* and *Globicatella* within this phylum were significantly higher in the RA group. The *Proteobacteria* phylum was not significantly different between RA cases and controls, but taxa in the class *Alphaproteobacteria*, *Betaproteobacteria* and *Gammaproteobacteria* showed significantly different. Besides, at genus level, *Ancylobacter* were presented with lower abundances among RA cases compared to those in controls, while the relative abundances of *Aureimonas*, *Devosia*, *Pelagibacterium*, *Chelativorans*, *Hoeflea*, *Burkholderia* and *Halomonas* were higher in RA cases than in healthy controls.

**Table S5 Bacterial taxa associated with RA related to gene functional pathways**

| **KEGG Pathway** | ***P* value** | **q value** |
| --- | --- | --- |
| Cellular Processes; Cell Growth and Death; Apoptosis | 3.35E-03 | 1.36E-02 |
| Cellular Processes; Cell Growth and Death; Meiosis - yeast | 5.91E-05 | 4.67E-04 |
| Cellular Processes; Cell Growth and Death; p53 signaling pathway | 1.75E-04 | 1.28E-03 |
| Cellular Processes; Cell Motility; Bacterial motility proteins | 1.15E-05 | 2.27E-04 |
| Cellular Processes; Cell Motility; Cytoskeleton proteins | 7.97E-03 | 2.72E-02 |
| Cellular Processes; Cell Motility; Flagellar assembly | 2.03E-06 | 5.62E-05 |
| Cellular Processes; Transport and Catabolism; Endocytosis | 2.38E-03 | 9.98E-03 |
| Cellular Processes; Transport and Catabolism; Peroxisome | 1.21E-06 | 1.67E-05 |
| Environmental Information Processing; Membrane Transport; ABC transporters | 2.83E-03 | 2.12E-02 |
| Environmental Information Processing; Membrane Transport; Bacterial secretion system | 8.59E-05 | 1.19E-03 |
| Environmental Information Processing; Membrane Transport; Phosphotransferase system (PTS) | 7.39E-03 | 4.99E-02 |
| Environmental Information Processing; Membrane Transport; Secretion system | 7.59E-05 | 1.11E-03 |
| Environmental Information Processing; Membrane Transport; Transporters | 9.49E-04 | 9.39E-03 |
| Environmental Information Processing; Signal Transduction; Phosphatidylinositol signaling system | 2.46E-09 | 1.70E-07 |
| Environmental Information Processing; Signaling Molecules and Interaction; Bacterial toxins | 4.23E-04 | 4.69E-03 |
| Environmental Information Processing; Signaling Molecules and Interaction; Cellular antigens | 3.72E-08 | 1.72E-06 |
| Environmental Information Processing; Signaling Molecules and Interaction; Ion channels | 6.29E-06 | 6.46E-05 |
| Genetic Information Processing; Folding, Sorting and Degradation; Chaperones and folding catalysts | 2.18E-03 | 1.72E-02 |
| Genetic Information Processing; Folding, Sorting and Degradation; Protein export | 3.07E-05 | 2.74E-04 |
| Genetic Information Processing; Replication and Repair; Chromosome | 9.23E-03 | 3.01E-02 |
| Genetic Information Processing; Replication and Repair; DNA replication | 3.80E-04 | 2.39E-03 |
| Genetic Information Processing; Replication and Repair; DNA replication proteins | 3.64E-03 | 1.46E-02 |
| Genetic Information Processing; Replication and Repair; Mismatch repair | 1.32E-11 | 3.66E-09 |
| Genetic Information Processing; Replication and Repair; Nucleotide excision repair | 5.21E-04 | 3.07E-03 |
| Genetic Information Processing; Transcription; RNA polymerase | 5.44E-03 | 2.01E-02 |
| Genetic Information Processing; Transcription; Transcription factors | 1.77E-08 | 1.64E-06 |
| Genetic Information Processing; Translation; Ribosome | 1.10E-04 | 8.43E-04 |
| Genetic Information Processing; Translation; Translation factors | 2.46E-04 | 1.70E-03 |
| Human Diseases; Cancers; Pathways in cancer | 3.95E-03 | 1.54E-02 |
| Human Diseases; Cancers; Renal cell carcinoma | 1.52E-03 | 7.26E-03 |
| Human Diseases; Cardiovascular Diseases; Hypertrophic cardiomyopathy (HCM) | 2.40E-06 | 3.02E-05 |
| Human Diseases; Infectious Diseases; Bacterial invasion of epithelial cells | 3.58E-07 | 7.62E-06 |
| Human Diseases; Infectious Diseases; Tuberculosis | 3.42E-04 | 2.20E-03 |
| Human Diseases; Neurodegenerative Diseases; Amyotrophic lateral sclerosis (ALS) | 2.18E-03 | 1.72E-02 |
| Human Diseases; Neurodegenerative Diseases; Huntington's disease | 1.67E-03 | 1.49E-02 |
| Human Diseases; Neurodegenerative Diseases; Prion diseases | 9.71E-05 | 1.28E-03 |
| Metabolism; Amino Acid Metabolism; Amino acid related enzymes | 2.83E-03 | 1.17E-02 |
| Metabolism; Amino Acid Metabolism; Arginine and proline metabolism | 1.75E-04 | 2.11E-03 |
| Metabolism; Amino Acid Metabolism; Cysteine and methionine metabolism | 1.07E-02 | 3.32E-02 |
| Metabolism; Amino Acid Metabolism; Histidine metabolism | 1.23E-04 | 9.24E-04 |
| Metabolism; Amino Acid Metabolism; Lysine biosynthesis | 5.91E-05 | 4.67E-04 |
| Metabolism; Amino Acid Metabolism; Phenylalanine metabolism | 5.03E-03 | 3.57E-02 |
| Metabolism; Amino Acid Metabolism; Phenylalanine, tyrosine and tryptophan biosynthesis | 4.60E-06 | 5.31E-05 |
| Metabolism; Amino Acid Metabolism; Valine, leucine and isoleucine biosynthesis | 4.70E-04 | 2.83E-03 |
| Metabolism; Amino Acid Metabolism; Valine, leucine and isoleucine degradation | 7.39E-03 | 4.99E-02 |
| Metabolism; Biosynthesis of Other Secondary Metabolites; Butirosin and neomycin biosynthesis | 5.44E-03 | 2.01E-02 |
| Metabolism; Biosynthesis of Other Secondary Metabolites; Caffeine metabolism | 1.44E-07 | 7.96E-06 |
| Metabolism; Biosynthesis of Other Secondary Metabolites; Novobiocin biosynthesis | 3.95E-03 | 1.54E-02 |
| Metabolism; Biosynthesis of Other Secondary Metabolites; Penicillin and cephalosporin biosynthesis | 9.23E-03 | 3.01E-02 |
| Metabolism; Biosynthesis of Other Secondary Metabolites; Phenylpropanoid biosynthesis | 3.51E-05 | 3.04E-04 |
| Metabolism; Biosynthesis of Other Secondary Metabolites; Stilbenoid, diarylheptanoid and gingerol biosynthesis | 8.42E-07 | 1.37E-05 |
| Metabolism; Biosynthesis of Other Secondary Metabolites; Streptomycin biosynthesis | 1.77E-07 | 4.91E-06 |
| Metabolism; Carbohydrate Metabolism; Amino sugar and nucleotide sugar metabolism | 1.44E-07 | 4.42E-06 |
| Metabolism; Carbohydrate Metabolism; Butanoate metabolism | 2.83E-03 | 2.12E-02 |
| Metabolism; Carbohydrate Metabolism; Glycolysis / Gluconeogenesis | 8.42E-07 | 1.37E-05 |
| Metabolism; Carbohydrate Metabolism; Inositol phosphate metabolism | 7.46E-08 | 5.17E-06 |
| Metabolism; Carbohydrate Metabolism; Propanoate metabolism | 1.01E-06 | 1.55E-05 |
| Metabolism; Carbohydrate Metabolism; Starch and sucrose metabolism | 5.88E-03 | 2.09E-02 |
| Metabolism; Energy Metabolism; Carbon fixation in photosynthetic organisms | 5.88E-03 | 2.09E-02 |
| Metabolism; Energy Metabolism; Carbon fixation pathways in prokaryotes | 1.67E-03 | 7.57E-03 |
| Metabolism; Energy Metabolism; Methane metabolism | 5.03E-03 | 1.91E-02 |
| Metabolism; Energy Metabolism; Nitrogen metabolism | 2.75E-04 | 1.81E-03 |
| Metabolism; Energy Metabolism; Oxidative phosphorylation | 1.67E-03 | 7.57E-03 |
| Metabolism; Energy Metabolism; Photosynthesis | 7.80E-04 | 4.00E-03 |
| Metabolism; Energy Metabolism; Photosynthesis proteins | 2.75E-04 | 1.81E-03 |
| Metabolism; Energy Metabolism; Sulfur metabolism | 4.01E-05 | 3.36E-04 |
| Metabolism; Enzyme Families; Peptidases | 1.15E-05 | 1.10E-04 |
| Metabolism; Glycan Biosynthesis and Metabolism; Glycosphingolipid biosynthesis - ganglio series | 1.82E-03 | 1.53E-02 |
| Metabolism; Glycan Biosynthesis and Metabolism; Glycosphingolipid biosynthesis - globo series | 1.23E-04 | 1.55E-03 |
| Metabolism; Glycan Biosynthesis and Metabolism; Glycosyltransferases | 9.92E-03 | 3.16E-02 |
| Metabolism; Glycan Biosynthesis and Metabolism; Peptidoglycan biosynthesis | 1.37E-08 | 7.58E-07 |
| Metabolism; Lipid Metabolism; Arachidonic acid metabolism | 1.14E-02 | 3.48E-02 |
| Metabolism; Lipid Metabolism; Fatty acid metabolism | 6.35E-03 | 2.23E-02 |
| Metabolism; Lipid Metabolism; Glycerolipid metabolism | 1.07E-02 | 3.32E-02 |
| Metabolism; Lipid Metabolism; Secondary bile acid biosynthesis | 1.82E-03 | 1.53E-02 |
| Metabolism; Lipid Metabolism; Sphingolipid metabolism | 5.95E-08 | 2.35E-06 |
| Metabolism; Lipid Metabolism; Synthesis and degradation of ketone bodies | 4.28E-03 | 1.65E-02 |
| Metabolism; Metabolism of Cofactors and Vitamins; Biotin metabolism | 5.39E-06 | 5.74E-05 |
| Metabolism; Metabolism of Cofactors and Vitamins; Folate biosynthesis | 1.31E-02 | 3.92E-02 |
| Metabolism; Metabolism of Cofactors and Vitamins; Lipoic acid metabolism | 1.71E-06 | 2.26E-05 |
| Metabolism; Metabolism of Cofactors and Vitamins; One carbon pool by folate | 9.23E-03 | 3.01E-02 |
| Metabolism; Metabolism of Cofactors and Vitamins; Retinol metabolism | 5.39E-06 | 5.74E-05 |
| Metabolism; Metabolism of Cofactors and Vitamins; Riboflavin metabolism | 3.26E-07 | 7.52E-06 |
| Metabolism; Metabolism of Cofactors and Vitamins; Vitamin B6 metabolism | 1.52E-03 | 7.26E-03 |
| Metabolism; Metabolism of Other Amino Acids; beta-Alanine metabolism | 3.34E-06 | 7.71E-05 |
| Metabolism; Metabolism of Other Amino Acids; Cyanoamino acid metabolism | 2.67E-07 | 6.72E-06 |
| Metabolism; Metabolism of Other Amino Acids; D-Alanine metabolism | 9.49E-04 | 4.78E-03 |
| Metabolism; Metabolism of Other Amino Acids; D-Arginine and D-ornithine metabolism | 5.88E-03 | 2.09E-02 |
| Metabolism; Metabolism of Other Amino Acids; D-Glutamine and D-glutamate metabolism | 5.81E-07 | 1.15E-05 |
| Metabolism; Metabolism of Other Amino Acids; Taurine and hypotaurine metabolism | 1.51E-02 | 4.39E-02 |
| Metabolism; Metabolism of Terpenoids and Polyketides; Biosynthesis of ansamycins | 7.07E-04 | 3.84E-03 |
| Metabolism; Metabolism of Terpenoids and Polyketides; Biosynthesis of siderophore group nonribosomal peptides | 4.23E-04 | 2.60E-03 |
| Metabolism; Metabolism of Terpenoids and Polyketides; Biosynthesis of vancomycin group antibiotics | 1.29E-09 | 1.19E-07 |
| Metabolism; Metabolism of Terpenoids and Polyketides; Carotenoid biosynthesis | 5.77E-04 | 3.26E-03 |
| Metabolism; Metabolism of Terpenoids and Polyketides; Limonene and pinene degradation | 9.92E-03 | 3.16E-02 |
| Metabolism; Metabolism of Terpenoids and Polyketides; Polyketide sugar unit biosynthesis | 1.29E-09 | 1.19E-07 |
| Metabolism; Metabolism of Terpenoids and Polyketides; Prenyltransferases | 1.15E-05 | 1.10E-04 |
| Metabolism; Metabolism of Terpenoids and Polyketides; Terpenoid backbone biosynthesis | 7.46E-08 | 2.58E-06 |
| Metabolism; Metabolism of Terpenoids and Polyketides; Zeatin biosynthesis | 5.77E-04 | 3.26E-03 |
| Metabolism; Nucleotide Metabolism; Pyrimidine metabolism | 7.80E-04 | 4.00E-03 |
| Metabolism; Xenobiotics Biodegradation and Metabolism; Benzoate degradation | 5.39E-06 | 1.15E-04 |
| Metabolism; Xenobiotics Biodegradation and Metabolism; Chloroalkane and chloroalkene degradation | 1.31E-02 | 3.92E-02 |
| Metabolism; Xenobiotics Biodegradation and Metabolism; Chlorocyclohexane and chlorobenzene degradation | 1.53E-05 | 2.50E-04 |
| Metabolism; Xenobiotics Biodegradation and Metabolism; Dioxin degradation | 2.83E-06 | 7.14E-05 |
| Metabolism; Xenobiotics Biodegradation and Metabolism; Drug metabolism - other enzymes | 1.21E-06 | 1.67E-05 |
| Metabolism; Xenobiotics Biodegradation and Metabolism; Metabolism of xenobiotics by cytochrome P450 | 9.23E-03 | 3.01E-02 |
| Metabolism; Xenobiotics Biodegradation and Metabolism; Nitrotoluene degradation | 1.51E-02 | 4.39E-02 |
| Metabolism; Xenobiotics Biodegradation and Metabolism; Polycyclic aromatic hydrocarbon degradation | 4.51E-09 | 1.25E-06 |
| Metabolism; Xenobiotics Biodegradation and Metabolism; Styrene degradation | 3.26E-07 | 1.13E-05 |
| Metabolism; Xenobiotics Biodegradation and Metabolism; Xylene degradation | 3.07E-05 | 4.73E-04 |

Bacterial gene functions were predicted from 16S ribosomal DNA gene-based microbial compositions using the PICRUSt (phylogenetic investigation of communities by reconstruction of unobserved states) algorithm, a computational approach to predict the functional composition of a metagenome using marker gene data and a database of reference genomes, to make inferences from the KEGG annotated databases. Functional analysis of the KEGG pathways related to 16S ribosomal DNA gene sequences detected 110 pathways that differed in abundance between the RA group and control group at FDR q values < 0.05.

**Table S6 Correlations between the identified blood bacterial taxa and methylation level in PBMCs**

| CpG site | CHR | Position* | Gene | Methylation-RA association | | | Bacteria ^#^ | Spearman correlation | |
| --- | --- | --- | --- | --- | --- | --- | --- | --- | --- |
|  |  |  |  | Mean RA | Mean control | *P* value |  | r | *P* value |
| cg00959259 | 3 | 122281975 | *PARP9* | 0.5664 | 0.6988 | 5.34E-03 | o_Rhizobiales | -0.6070 | 1.66E-03 |
| cg00959259 | 3 | 122281975 | *PARP9* | 0.5664 | 0.6988 | 5.34E-03 | f_Hyphomicrobiaceae | -0.5539 | 4.98E-03 |
| cg00959259 | 3 | 122281975 | *PARP9* | 0.5664 | 0.6988 | 5.34E-03 | g_Pelagibacterium | -0.5626 | 4.21E-03 |
| cg22274273 | 6 | 135137590 | *-* | 0.6212 | 0.3774 | 4.02E-03 | f_Hyphomonadaceae | -0.5362 | 6.91E-03 |
| cg23785719 | 7 | 43622659 | *STK17A* | 0.3556 | 0.5259 | 1.19E-04 | f_Hyphomonadaceae | 0.5870 | 2.57E-03 |
| cg13444538 | 7 | 158905317 | *VIPR2* | 0.6989 | 0.5973 | 7.01E-03 | o_Rhodospirillales | 0.5309 | 7.60E-03 |
| cg13444538 | 7 | 158905317 | *VIPR2* | 0.6989 | 0.5973 | 7.01E-03 | f_Rhodospirillaceae | 0.5309 | 7.60E-03 |

* Assembly: GRCh37.p13.

^#^ ‘o_’ represented order; ‘f_’ represented family; ‘g_’ represented genus.

**Table S7 Correlations between the identified blood bacterial taxa and mRNA expression levels in PBMCs**

| Probe name | Gene | mRNA-RA association | | | Bacteria ^#^ | Spearman correlation | |
| --- | --- | --- | --- | --- | --- | --- | --- |
|  |  | Fold change | *P* value | Regulation |  | r | *P* value |
| A_21_P0013755 | *FAM3C* | 3.32 | 2.92E-06 | UP | g_Pelagibacterium | 0.8035 | 2.27E-06 |
| A_21_P0013755 | *FAM3C* | 3.32 | 2.92E-06 | UP | f_Hyphomicrobiaceae | 0.7991 | 2.83E-06 |
| A_24_P38276 | *FZD1* | 2.34 | 5.45E-07 | UP | f_Hyphomonadaceae | -0.7471 | 2.73E-05 |
| A_21_P0013755 | *FAM3C* | 3.32 | 2.92E-06 | UP | o_Rhizobiales | 0.7470 | 2.75E-05 |
| A_23_P393425 | *PAPD4* | 2.11 | 2.80E-06 | UP | f_Hyphomicrobiaceae | 0.7113 | 9.75E-05 |
| A_23_P364107 | *MIS18BP1* | 2.11 | 4.51E-05 | UP | g_Saccharibacteria genera incertae sedis | -0.7078 | 1.09E-04 |
| A_23_P90612 | *MCM6* | 2.08 | 6.51E-05 | UP | g_Pelagibacterium | 0.7078 | 1.09E-04 |
| A_23_P363647 | *DDX26B* | 2.33 | 1.82E-04 | UP | f_Hyphomicrobiaceae | 0.6983 | 1.48E-04 |
| A_23_P90612 | *MCM6* | 2.08 | 6.51E-05 | UP | f_Hyphomicrobiaceae | 0.6974 | 1.52E-04 |
| A_23_P363647 | *DDX26B* | 2.33 | 1.82E-04 | UP | g_Pelagibacterium | 0.6939 | 1.69E-04 |
| A_33_P3299872 | *HINT3* | 2.78 | 2.31E-06 | UP | o_Rhizobiales | 0.6904 | 1.88E-04 |
| A_23_P393425 | *PAPD4* | 2.11 | 2.80E-06 | UP | g_Pelagibacterium | 0.6896 | 1.93E-04 |
| A_23_P146187 | *RRS1* | 2.01 | 2.41E-06 | UP | f_Aurantimonadaceae | 0.6880 | 2.02E-04 |
| A_24_P288424 | *SLMO2* | 2.39 | 6.58E-06 | UP | f_Aurantimonadaceae | 0.6784 | 2.69E-04 |
| A_33_P3299872 | *HINT3* | 2.78 | 2.31E-06 | UP | g_Pelagibacterium | 0.6783 | 2.70E-04 |
| A_33_P3299872 | *HINT3* | 2.78 | 2.31E-06 | UP | f_Hyphomicrobiaceae | 0.6730 | 3.13E-04 |
| A_21_P0000015 | *PARP9* | 3.86 | 1.58E-07 | UP | f_Hyphomicrobiaceae | 0.6632 | 4.12E-04 |
| A_33_P3303697 | *CR2* | 2.18 | 2.00E-04 | DOWN | f_Hyphomonadaceae | 0.6588 | 4.64E-04 |
| A_21_P0000015 | *PARP9* | 3.86 | 1.58E-07 | UP | g_Pelagibacterium | 0.6549 | 5.15E-04 |
| A_24_P926507 | *SLC14A1* | 2.49 | 7.32E-05 | UP | f_Hyphomicrobiaceae | 0.6539 | 5.29E-04 |
| A_24_P926507 | *SLC14A1* | 2.49 | 7.32E-05 | UP | g_Pelagibacterium | 0.6452 | 6.63E-04 |
| A_24_P226210 | *CEP120* | 2.22 | 5.81E-06 | UP | f_Hyphomicrobiaceae | 0.6435 | 6.93E-04 |
| A_21_P0013755 | *FAM3C* | 3.32 | 2.92E-06 | UP | f_Aurantimonadaceae | 0.6414 | 7.31E-04 |
| A_24_P226210 | *CEP120* | 2.22 | 5.81E-06 | UP | g_Pelagibacterium | 0.6383 | 7.91E-04 |
| A_23_P50108 | *NDC80* | 2.26 | 1.26E-05 | UP | g_Pelagibacterium | 0.6365 | 8.26E-04 |
| A_24_P108291 | *IMPACT* | 2.44 | 1.39E-05 | UP | g_Saccharibacteria genera incertae sedis | -0.6330 | 9.00E-04 |
| A_23_P50108 | *NDC80* | 2.26 | 1.26E-05 | UP | f_Hyphomicrobiaceae | 0.6304 | 9.59E-04 |

^#^ ‘p_’ represented phylum; ‘c_’ represented class; ‘o_’ represented order; ‘f_’ represented family; ‘g_’ represented genus.

For the mRNA expression data, correlations were detected between bacteria within *Candidatus Saccharibacteria* phylum and *Proteobacteria* phylum and mRNA expression levels in PBMCs. A total of 27 correlations with *P* < 1.0×10^-3^ were found.

**Table S8 Causal inference test results for RA risk influenced by bacterial taxa through mediation of *PARP9* mRNA level**

| **Bacterium** | ***P*_bacteria_RA^*^** | ***P*_adj_RA ^¶^** | ***P*_adj_bacteria ^†^** | ***P_*adj_mRNA ^‡^** |
| --- | --- | --- | --- | --- |
| Order Rhizobiales | 8.42E-07 | 4.91E-02 | 2.29E-05 | 0.14 |
| Family Hyphomicrobiaceae | 1.44E-07 | 4.36E-02 | 6.20E-05 | 0.11 |
| Genus Pelagibacterium | 9.32E-08 | 4.39E-02 | 6.60E-05 | 0.11 |

Further, we performed in-depth causal inference test to explore whether the identified bacterial taxa affect RA through regulating DNA methylation or gene expression, i.e., to assess the potential regulatory chain of causal factor (blood bacteria)–mediator (DNA methylation or mRNA levels in PBMCs)–outcome (RA). Through causal inference test using data from the 28 RA patients and 15 controls, we detected causal effects of order *Rhizobiales*, family *Hyphomicrobiaceae* and genus *Pelagibacterium* and the mediation effect of *PARP9* mRNA expression on RA risk.

The causal inference test is a model selection approach based on conditional correlation, in which causality can be inferred if four conditions are met:

Condition 1: Bacteria and RA were associated;

Condition 2: Bacteria were associated with *PARP9* mRNA level after adjusting for RA;

Condition 3: *PARP9* mRNA level was associated with RA after adjusting for bacteria;

Condition 4: Bacteria were independent of RA level after adjusting for *PARP9* mRNA.

*: Crude *P* value for the association of bacteria with RA. Condition 1 was met if this *P* value was less than 0.05;

¶: *P* value for the association of bacteria with *PARP9* mRNA level after adjusted for RA. Condition 2 was met if this *P* value was less than 0.05;

†: *P* value for the association of *PARP9* mRNA level with RA after adjusted for bacteria. Condition 3 was met if this *P* value was less than 0.05;

‡: *P* value for the association of bacteria with RA after adjusted for *PARP9* mRNA level. Condition 4 was met if this *P* value was greater than 0.05.
